# Machine learning based method for analyzing vibration and noise in large cruise ships

**DOI:** 10.1371/journal.pone.0307835

**Published:** 2024-07-25

**Authors:** Wenwei Wu, Tao He, Xiaying Hao, Kaiwei Xu, Ji Zeng, Jiahui Gu, Lei Chen

**Affiliations:** 1 China Ship Scientific Research Center, Wuxi, Jiangsu Province, China; 2 College of Ocean Science and Engineering, Shanghai Maritime University, Shanghai, China; 3 College of Information Engineering, Shanghai Maritime University, Shanghai, China; Aalto University, FINLAND

## Abstract

Cruise ships are distinguished as special passenger ships, transporting passengers to various ports and giving importance to comfort. High comfort can attract lots of passengers and generate substantial profits. Vibration and noise are the most important indicators for assessing the comfort of cruise ships. Existing methods for analyzing vibration and noise data have shown limitations in uncovering essential information and discerning critical disparities in vibration and noise levels across different ship districts. Conversely, the rapid development in machine learning present an opportunity to leverage sophisticated algorithms for a more insightful examination of vibration and noise aboard cruise ships. This study designed a machine learning-driven approach to analyze the vibration and noise data. Drawing data from China’s first large-scale cruise ship, encompassing 127 noise samples, this study sets up a classification task, where decks were assigned as labels and frequencies served as features. Essential information was extracted by investigating this problem. Several machine learning algorithms, including feature ranking, selection, and classification algorithms, were adopted in this method. One or two essential noise frequencies related to each of the decks, except the 10th deck, were obtained, which were partly validated by the traditional statistical methods. Such findings were helpful in reducing and controlling the vibration and noise in cruise ships. Furthermore, the study develops a classifier to distinguish noise samples, which utilizes random forest as the classification algorithm with eight optimal frequency features identified by LightGBM. This classifier yielded a Matthews correlation coefficient of 0.3415. This study gives a new direction for investigating vibration and noise in ships.

## Introduction

Cruise ships are large passenger ships that are mainly used for vacationing. Generally, they carry passengers to various ports, where passengers can go ashore for a simple tour. On the ships, passengers can receive several services, such as entertainments, sports, and catering. The comfort of cruise ships is one of the most important indicators when designing and building the ships. A cruise ship with excellent comfort can attract more and more passengers, thereby bringing lots of profits. Comfort is the lifeline of cruise ships, especially for large cruise ships due to the large initial investment. Several aspects measure the comfort of cruise ships, including space layout, color and light environment, vibration and noise, and air environment [1]. Among them, vibration and noise are the most important aspects because they constantly influence passengers. Although two different concepts, they have a close relationship. In detail, noise is usually produced by vibration. An effective method for controlling noise is to reduce the vibration amplitude of the vibration source. Thus, they are always investigated together. Some leading classification societies, such as Lloyd’s Register of Shipping, Registro Italiano Navale, Det Norske Veritas, and Bureau Veritas propose strict requirements on the noise of cruise ships. Thus, designing effective methods for the analysis of vibration and noise in cruise ships is necessary and urgent. Based on the results of the analysis, the managers can design targeted schemes to reduce and control vibration and noise, improving the comfort of cruise ships.

Given several difficulties in building a large cruise ship, few counties and shipyards can independently complete the whole procedure. Thus, few materials can be referred to for investigating the vibration and noise in cruise ships because a few people control these materials. Accordingly, studies on vibration and noise in cruise ships are few. Empirical formula and numerical analysis are two major traditional methods for analyzing vibration and noise data on ships. Evidently, the empirical formula is not a reliable method because experiences are not always correct. Numerical analysis methods are more popular at present. It includes statistical energy analysis (SEA) [2–4], finite element analysis (FEA) [5, 6], the combination of SEA and FEA [7–10], and other methods [11, 12]. The numerical methods significantly promotes the development of the research on vibration and noise in ships. These methods mainly focused on analyzing the sources of vibration and noise and establishing forecasting methods to simulate or recover noise data. To our knowledge, few studies investigate the essential differences in vibration and noise among different districts of a ship. To fulfill this purpose, a deep investigation of vibration and noise data is imperative to uncover essential information within the dataset. The general statistical and numerical methods are not powerful enough to mine this essential information. In recent years, many machine learning algorithms have been designed and successfully applied to tackle various practical problems [13, 14]. Their mining ability is much more potent than traditional numerical methods. Their application in the analysis of vibration and noise data is a new and interesting direction.

**Fig 1:**
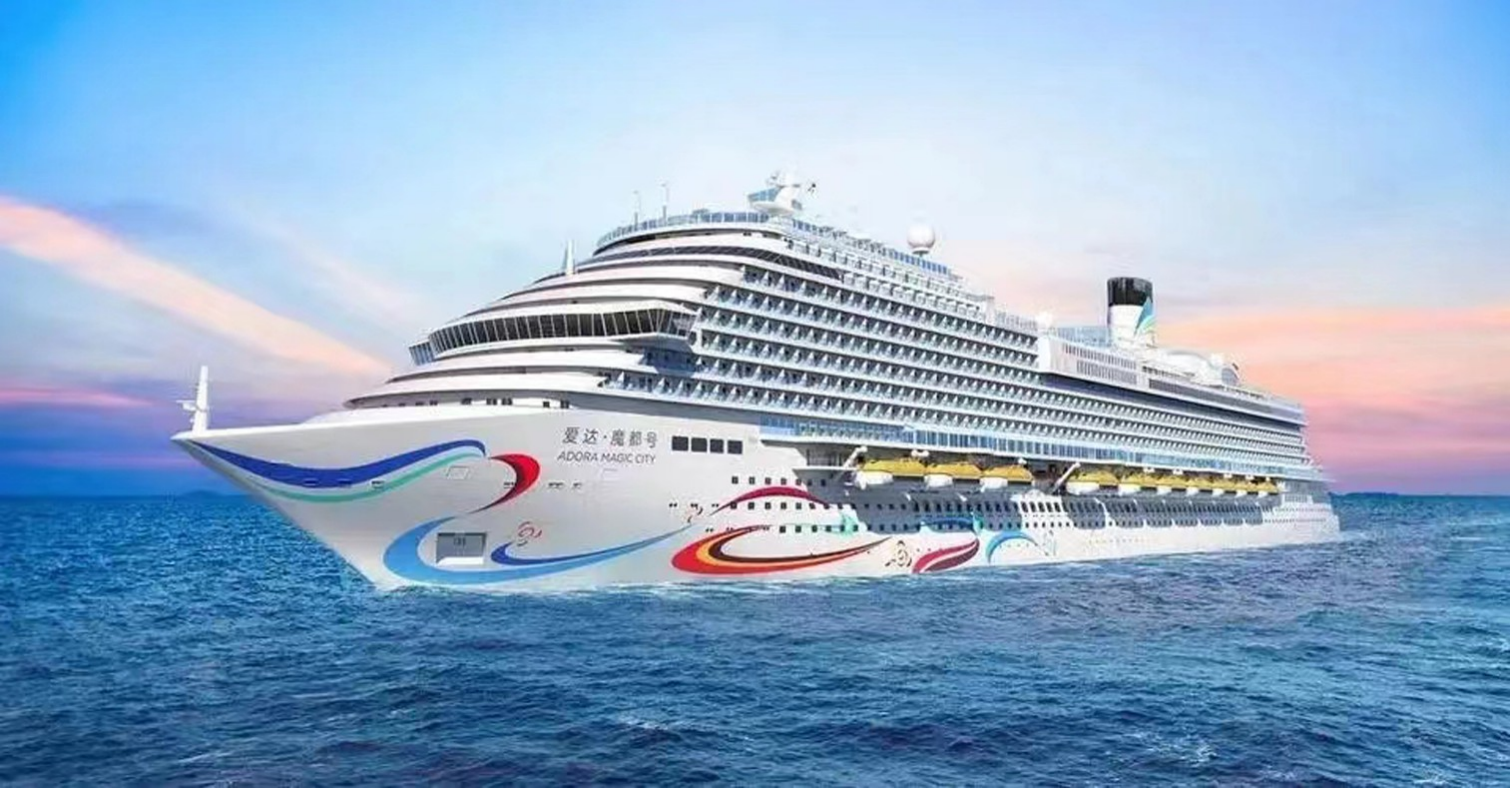
The first large cruise ship made in China, named Adora Magic City.

This study designed a machine learning-based method for analyzing vibration and noise in the first cruise ship made in China (**Fig 1)**. The method contained several machine learning algorithms. Different from previous numerical methods designed for ship cabins, this study was conducted for the decks in large cruise ships. Generally, a large cruise ship always contains several decks. The vibration and noise on different decks may have essential differences. Thus, the purpose of this study was to uncover the critical differences in vibration and noise on different decks. The decks were deemed labels, and frequencies were regarded as features, thereby modeling a classification problem. The features were first analyzed by three feature ranking algorithms [15–17]. Then, the label-removing test followed to evaluate the relationships between frequencies and decks. As a result, some frequencies were obtained for some decks, which were considered to have strong associations with corresponding decks. Statistical methods further validated this result. Furthermore, the results of feature ranking algorithms were fed into the incremental feature selection (IFS) [18], incorporating random forest (RF) [19] as the classification algorithm to build an efficient classifier for distinguishing noise samples. This study provided an alternative way to investigate vibration and noise in cruise ships.

The following text is organized as follows. “Materials and Methods” describes the investigated dataset, computational methods, and performance measurements. “Results and Discussion” gives the computational results and analyzes the reliability of the results. The final section presents the conclusion of this study.

## Materials and methods

### Dataset

The data on vibration and noise was collected from a newly built cruise ship (named Adora Magic City) in July 2023, which is the first large cruise ship made in China. There are 12 decks on this ship. The RION NA-28, a type of sound level meter, was placed in 127 different locations, which were distributed from the third deck to the twelfth deck. Thus, 127 samples can be obtained. The number of samples in each deck is listed in **Table 1**. The samples for the third and sixth decks were the most (23), whereas the eighth deck had the least samples (3). According to the theory of the one-third octave band, the frequency can be divided into several intervals. The center frequency in each interval is usually adopted to measure noise. As the frequency of audible noise is less than 20 kHz, 43 intervals can be obtained using a one-third octave band. After removing the intervals with small and large frequencies, we finally selected 31 intervals, and their center frequency was used to decompose each noise sample. That is, each noise sample was decomposed into 31 center frequencies, which are provided in the S1 Table. The values of the above 31 center frequencies for a noise sample can directly be retrieved from RION NA-28. Based on this vibration and noise dataset, this study aims to analyze the essential differences of frequencies on different decks, providing new ways to investigate the vibration and noise in large cruise ships.

**Table 1:** Breakdown of the vibration and noise data.

Index	Label	Deck	Number of samples
1	*L*_3_	Third deck	23
2	*L*_4_	Fourth deck	11
3	*L*_5_	Fifth deck	13
4	*L*_6_	Sixth deck	23
5	*L*_7_	Seventh deck	10
6	*L*_8_	Eighth deck	3
7	*L*_9_	Ninth deck	6
8	*L*_10_	Tenth deck	16
9	*L*_11_	Eleventh deck	18
10	*L*_12_	Twelfth deck	4
Total			127

### Problem description

The traditional means for analyzing the noise frequency spectrum always rely on knowledge of statistics. Here, we proposed a novel scheme to analyze such frequency spectrum. A classification problem was set up, and the further analysis was conducted on this problem. As mentioned in Section “Dataset,” the noise samples were obtained from 10 different decks in Adora Magic City. When building the classification problem, these decks were deemed as labels, denoted by *L*_3_, *L*_4_, ⋯, *L*_12_, where the subscript denoted the deck. The 31 frequencies were considered as features, formulated as *F*_1_, *F*_2_, ⋯, *F*_31_. Accordingly, the investigated dataset was formulated as {(*s*_*i*_, *l*_*i*_)|*i* = 1,2, ⋯, 127}, where *s*_*i*_ represented the *i*-th noise sample, *l*_*i*_∈{*L*_3_, *L*_4_, ⋯, *L*_12_} denoted the deck where the *i*-th noise sample was obtained. Furthermore, each sample *s*_*i*_ was represented by a 31-D (dimension) vector formulated by

$$V\left(s_{i}\right)={\left[f_{i}^{1},f_{i}^{2},\cdots ,f_{i}^{31}\right]}^{T},$$ (1)

where $f_{i}^{j}$ indicated the value of *s*_*i*_ under the feature *F*_*j*_ (the decomposition value under one frequency). By analyzing this classification problem, some essential features can be extracted, and the features highly related to a certain deck can be obtained.

### Feature ranking algorithms

In machine learning, several algorithms have been proposed to extract important features that can provide critical contributions to classification. These algorithms always sort features in terms of their importance. In general, each algorithm has advantages and disadvantages. Thus, one algorithm can only mine a part of important features. Using multiple algorithms is a feasible way to tackle this problem. In this study, three feature ranking algorithms were employed, including a light gradient boosting machine (LightGBM) [15], Extreme Gradient Boosting (XGBoost) [16], and Categorical Boosting (CATBoost) [17]. These algorithms are designed using different principles and can overview the investigated dataset from various points of view, which is helpful in discovering important features. A brief description of these algorithms is discussed below.

#### Light gradient boosting machine

Gradient boosting decision tree (GBDT) [20] is an ensemble learning algorithm that constructs a more robust prediction model by combining multiple decision trees. GBDT excels in handling complex nonlinear relationships, diverse feature types, and robustness against outliers. However, given its sequential nature, the efficiency and accuracy of GBDT can be challenging to satisfy, especially when dealing with a large number of samples or high-dimensional feature spaces.

The primary cost during the learning process of GBDT is focused on finding the optimal split points for decision trees. To enhance efficiency, Ke et al. [15] proposed an efficient GBDT algorithm known as LightGBM. It employs gradient-based one-side sampling (GOSS) and exclusive feature bundling (EFB) techniques to improve efficiency.

LightGBM employs GOSS to determine the split points by calculating variance gain. Initially, the absolute values of the gradients of training examples are sorted in descending order, and the top *a*×100% data samples with the highest gradient values are selected, referred to as *A*. Subsequently, a subset *B* is randomly chosen from the retained samples *A*^*c*^ with a size of *b*×|*A*^*c*^|. Finally, instances are split based on the estimated variance gain ${\tilde{V}}_{j}(d)$ over the subset *A*∪*B*.

$${\tilde{V}}_{j}(d)=\frac{1}{n}\left(\frac{{\left(\sum_{x_{i}\in A_{l}}g_{i}+\frac{1-a}{b}\sum_{x_{i}\in B_{l}}g_{i}\right)}^{2}}{n_{l}^{j}(d)}+\frac{{\left(\sum_{x_{i}\in A_{r}}g_{i}+\frac{1-a}{b}\sum_{x_{i}\in Br}g_{i}\right)}^{2}}{n_{r}^{j}(d)}\right),$$ (2)

where $A_{l}=\left\{x_{i}\in A:x_{ij}\leq d\right\}$, $A_{r}=\left\{x_{i}\in A:x_{ij}>d\right\}$, $B_{l}=\left\{x_{i}\in B:x_{ij}\leq d\right\}$, $B_{r}=\left\{x_{i}\in B:x_{ij}\leq d\right\}$, *g*_*i*_ represents the negative gradient of the loss function, and $\frac{1-a}{b}$ is utilized to normalize the sum of gradients.

In high-dimensional features, many sparse features may be mutually exclusive, implying that certain features in a given sample might almost simultaneously be 0. EFB can bundle these mutually exclusive features together, forming a new feature, thereby reducing the dimensionality and sparsity of the data and improving training efficiency. Therefore, EFB can accelerate GBDT training.

In summary, LightGBM is an efficient implementation of GBDT and can more effectively handle high-dimensional, sparse feature data. LightGBM enhances computational efficiency without compromising accuracy by adopting GOSS and EFB. GOSS is used to split optimal nodes by calculating variance gain. Meanwhile, EFB bundles many mutually exclusive features into fewer dense features, accelerating the training process of GBDT.

This study adopted the LightGBM program, which can be accessed at https://lightgbm.readthedocs.io/en/stable/. It was executed with default parameters.

#### Extreme gradient boosting

XGBoost [16] is a sophisticated machine-learning algorithm proposed by Chen and Guestrin in 2011. Positioned as a boosting tree model, XGBoost represents a learning framework that has become a cornerstone in predictive modeling. In contrast to traditional boosting tree models that rely solely on first derivative information, XGBoost introduces a second-order Taylor expansion on the loss function during training. This unique approach facilitates the automatic utilization of CPU multithreading for efficient parallel computing. A distinguishing feature of XGBoost is its ability to address the challenge of distributed training encountered in models relying on the residual information from the former *n*-1 trees. Furthermore, XGBoost incorporates a suite of techniques aimed at preventing overfitting, making it a robust and versatile tool for a diverse range of machine-learning tasks. The model’s evolution since its inception highlights its adaptability and continuous refinement, solidifying its status as a robust algorithm in the machine-learning landscape.

XGBoost combines tree models through an additive method. Assuming there are *K* trees in total and uses *F* to denote the base tree model, then

$${\hat{y}}_{i}=\sum_{k=1}^{K}f_{k}\left(x_{i}\right),f_{k}\in F.$$ (3)

The objective function it employs is as follows:

$$L=\sum_{i}\;l\left({\hat{y}}_{i},y_{i}\right)+\sum_{k}\;\Omega \left(f_{k}\right),$$ (4)

where *l* is the loss function, representing the error between the true values and the predicted values, and Ω is a regularization function used to prevent overfitting. The Ω function is defined as follows:

$$\Omega (f)=\gamma T+\frac{1}{2}\lambda ∥w{∥}^{2},$$ (5)

where *T* represents the number of leaves in each tree and *w* represents the weights of the leaves in each tree.

After the second-order Taylor expansion of the objective function and a series of other calculations, the information gain for each split of the objective function can be obtained as follows:

$$\text{Gain}=\frac{1}{2}\left[\frac{{\left(\sum_{i\in I_{L}}g_{i}\right)}^{2}}{\sum_{i\in I_{L}}h_{i}+\lambda }+\frac{{\left(\sum_{i\in I_{R}}g_{i}\right)}^{2}}{\sum_{i\in I_{R}}h_{i}+\lambda }+\frac{{\left(\sum_{i\in I}g_{i}\right)}^{2}}{\sum_{i\in I}h_{i}+\lambda }\right]-\gamma ,$$ (6)

where *γ* is the split threshold and leaf node splitting is allowed only when the information gain is greater than *γ*. This mechanism is introduced to control the growth of the tree and prevent overfitting of the model.

In summary, XGBoost is a robust gradient-boosting algorithm based on ensemble learning with decision trees. It is optimized by combining the loss function and regularization term and employing a split threshold to control tree growth and prevent overfitting. With parallel computation and feature importance assessment, XGBoost excels in various machine-learning tasks, showcasing efficiency, flexibility, and robust performance.

The XGBoost program used in this study was obtained from https://xgboost.readthedocs.io/en/stable/, which was performed using default parameters.

#### Categorical boosting

CATBoost [17] is an effective machine-learning algorithm for predicting categorical features. It is an implementation of gradient boosting that utilizes binary decision trees as fundamental prediction factors. CATBoost excels in rapidly processing non-numeric features. When dealing with categorical features, the CATBoost algorithm incorporates the entire dataset for learning. Subsequently, it randomly permutes all samples and selects samples with the same category from all features. During the numerical transformation of each sample’s features, it first calculates the target value for the sample, adds corresponding weights, and assigns priorities. The specific formula is as follows:

$$x_{k}^{i}=\frac{\sum_{j=1}^{n}\left\{x_{j}^{i}=x_{k}^{i}\right\}\cdot y_{i}+ap}{\sum_{j=1}^{n}\left\{x_{j}^{k}=x_{k}^{i}\right\}+a},$$ (7)

where the prior value *p* and parameter *a* > 0 serve as weights, aiding in reducing noise obtained from low-frequency categories. This formula effectively mitigates model overfitting, thereby enhancing generalization capability.

In summary, CATBoost is a robust machine-learning algorithm designed specifically for handling categorical features. It belongs to the gradient-boosting algorithm family and utilizes binary decision trees as the fundamental building blocks for predictions. Notably, CATBoost stands out for its efficient handling of non-numeric features without the need for explicit encoding. When dealing with categorical features, the algorithm incorporates the entire dataset for learning, randomly permuting samples and selecting those with the same category from all features. Additionally, CATBoost introduces a prior weighting mechanism, which aids in reducing noise from low-frequency categories and mitigating overfitting. CATBoost is widely recognized for its robust performance across various machine learning tasks, especially when the datasets contain complex categorical features.

This study adopted the CATBoost program retrieved from https://catboost.ai/en/docs/. For convenience, it was executed with its default parameters.

The above three feature ranking algorithms were applied to the investigated dataset, generating three feature lists. For convenience, these lists were called LightGBM, XGBoost, and CATBoost feature lists. This study adopted public programs to execute these algorithms. In detail, the LightGBM program was obtained from https://lightgbm.readthedocs.io/en/latest/, the XGBoost program was retrieved from https://xgboost.readthedocs.io/en/stable/, and the CATBoost program was downloaded at https://catboost.ai/en/docs/. All programs were performed with default parameters, where LightGBM and XGBoost used the split times of features to assess the importance of features, whereas CATBoost adopted the change on the performance when the values under a feature were permuted to evaluate the importance of features.

### Label removing test for extracting important features related to one label

The above three feature ranking algorithms can only assess the importance of features for classification. Knowing which features are highly related to a certain label (i.e., deck in this study) remains a problem. Thus, a novel scheme was designed on the basis of the above three feature ranking algorithms. We called this scheme the label-removing test.

In general, if one feature is highly related to one label, its importance is also strongly dependent on this label and the samples with this label. Thus, a hypothesis can be concluded. When the samples with this label were removed from the classification problem, the importance of this feature would be reduced. Reflected by the feature list yielded by one feature ranking algorithm, the rank of this feature would decrease. The feature with a large decrease in rank has stronger relationships to the removed label than that with a small decrease. Accordingly, we evaluated the relationships of each feature to one label in the following manner. Given a label *L_i_* and feature *F_j_*, the rank of *F_j_* in the feature list by applying one feature ranking algorithm to the entire dataset is denoted by *R*(*F*_*j*_). The samples with label *L*_*i*_ are removed to generate a new dataset, which is fed into the same feature ranking algorithm. A new feature list is generated and the rank of *F*_*j*_ in this list is represented by $R_{L_{i}}\left(F_{j}\right)$. The gap between these two ranks can be calculated as follows:

$$G_{L_{i}}\left(F_{j}\right)=R\left(F_{j}\right)-R_{L_{i}}\left(F_{j}\right).$$ (8)

Evidently, the smaller the outcome of **Eq 8**, the stronger the relationships between feature *F*_*j*_ and label *L*_*i*_. For one label *L*_*i*_, all features can be ranked in a list based on the outcome of **Eq 8**. Features with high ranks are deemed to have strong relationships to the label *L*_*i*_. The essential features can be picked up from this list by setting a threshold *T*∈(0,1) to this list, which represents the proportion of features. In other words, the top *m* × *T* features in the list are selected, where *m* is the total number of features.

Three feature ranking algorithms were employed in this study to test the importance of features fully. Thus, for one label *L*_*i*_, three feature lists can be obtained. After setting a unified threshold, some important features can be accessed from each list. The intersection operation is conducted to select the most related features for the label *L*_*i*_.

### Outline of the machine learning-based method

**Fig 2:**
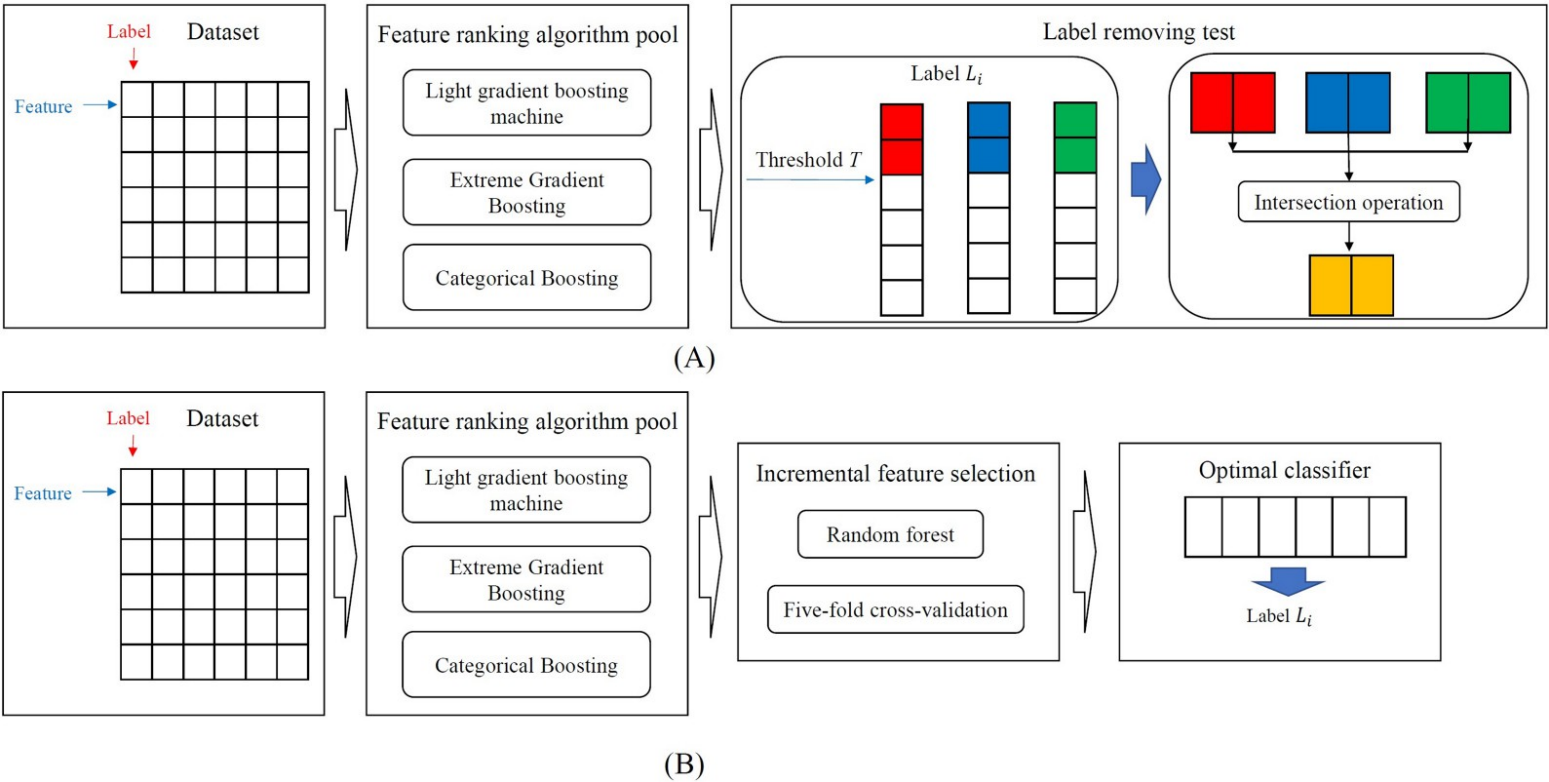
Entire procedure of this study. (A) Procedure of the machine learning based method for extracting important noise frequencies related to one deck. The dataset collected from Adora Magic City is investigated, where decks are deemed as labels and frequencies are regarded as features. Three feature ranking algorithms are used to evaluate the importance of features and the label removing test is designed to extract important features related to one label. (B) Procedure for building the optimal classifier. The feature lists yielded by three feature ranking algorithms are fed into incremental feature selection, incorporating random forest as the classification algorithm, to construct the optimal classifier.

In this study, a machine learning-based method was designed to analyze the vibration and noise data on cruise ships. The entire procedure is illustrated in **Fig 2(A)**. First, we modeled this analysis problem as a classification problem, where decks were deemed as labels and frequencies were regarded as features. Then, three feature-ranking algorithms were employed to analyze the data and further used in the label-removing test. This test ranked features according to their relationships to each label and discovered important features from the results of one feature ranking algorithm by setting a threshold *T*. Finally, the most related features were obtained using the intersection operation on the important features discovered by three feature ranking algorithms.

### Incremental feature selection

In addition to extracting features related to one label, a classifier can also be constructed using feature lists yielded by three feature-ranking algorithms. Given that not all features contribute positively to the classification process, carefully selecting the features to be included in the classification model is imperative. Here, the IFS [18] method was adopted to select such features. Given a feature list, IFS constructs a series of feature subsets, where the first subset contains the top feature in the list, the second subset consists of the top two features in the list, and so forth. Samples are represented by features in each feature subset, and a classifier is built on these samples using a given classification algorithm. The performance of this classifier is evaluated by the cross-validation method [21]. After all classifiers have been assessed, the classifier with the best performance is picked up as the optimal classifier.

### Classification algorithm

According to the IFS procedure, one classification algorithm is necessary. This study selected the classic and powerful RF [19], which has wide applications in constructing efficient classifiers [22–30]. In fact, RF is an integrated classification algorithm that contains several decision trees. The construction of each decision tree is well-designed. First, the samples for constructing each decision tree are randomly selected, with replacements, from the training dataset, and the selected samples are as many as those in the training dataset. Second, the decision tree is grown by randomly selecting a part of the features and selecting the optimal splitting on these features. These two random selections overcome the overfitting problem of a decision tree. After the predefined number of decision trees is set up, the RF integrates them using majority voting. In detail, for a query sample, each decision tree provides the predicted class. The class with the most votes is the outcome of RF. This study used the RF program in Scikit-learn [31]. Its main parameter, the number of decision trees, was set to 150.

### Performance evaluation

The classifiers built in the IFS method were evaluated by the cross-validation method [21]. In this method, the training samples are randomly and equally divided into *K* parts. Each part is singled out one by one as the test dataset, whereas the rest of the parts constitute the training dataset. The classifier built on the training dataset is applied to the test dataset to evaluate its performance. The average performance on all parts is calculated as the final performance of the classifier. Generally, *K* is set to five or ten. In this study, we selected five-fold cross-validation (i.e., *K* was set to five).

For a multi-class classification, accuracy is the most widely used measurement to evaluate the quality of predicted results. It is defined as the proportion of correctly predicted samples to all samples. That is,

$$\text{Accuracy}=\frac{P}{N},$$ (9)

where *P* is the number of correctly predicted samples and *N* is the total number of samples. Moreover, according to the distribution of samples in ten classes (cf. **Table 1**), sample numbers in different classes vary in an extensive range; that is, the dataset is imbalanced. In this case, accuracy is not a perfect measurement. Matthews correlation coefficient (MCC) [32] is a more proper measurement. However, the original MCC was designed for binary classification. Gorodkin extended the original MCC to multi-class [33]. To compute MCC in multi-class, two matrices should be constructed first, say *X* and *Y*, where *X* stores the true class of each sample and *Y* collects the predicted classes of samples. Then, MCC in multi-class can be computed by

$$MCC=\frac{cov(X,Y)}{\sqrt{cov(X,X)\cdot cov(Y,Y)}},$$ (10)

where *cov* represents the covariance of two matrixes. This study adopted MCC in multi-class as the key measurement. For easy description, it was still called MCC.

Furthermore, we employed macro precision and macro recall to evaluate the performance of classifiers. The precision and recall for each class should be calculated in advance. For the *i*-th class, samples belonging to this class are set as positive samples, whereas other samples are regarded as negative samples. According to the predicted results, recall and precision for this class can be computed as follows:

$$Recall_{i}=\frac{TP}{TP+FN},$$ (11)

$$precision_{i}=\frac{TP}{TP+FP},$$ (12)

where *TP* is true positives, *FN* is false negatives, and *FP* is false positives. Then, macro precision and macro recall can be calculated as follows:

$$Macro\;recall=\frac{1}{L}\sum_{i=1}^{L}\;Recall_{i},$$ (13)

$$Macro\;precision=\frac{1}{L}\sum_{i=1}^{L}\;precision_{i},$$ (14)

where *L* is the number of classes. Macro recall (precision) is the average of recall (precision) in all classes, which can evaluate the overall performance of classifiers.

## Results and discussion

In this study, a machine learning-based method was designed to analyze the vibration and noise data on cruise ships. Its procedure is displayed in **Fig 2(A)**. Furthermore, we also want to build a classifier to classify noise samples into corresponding decks correctly. The construction procedure can be found in **Fig 2(B)**. This section provided detailed results of the above procedures and elaborated on their reasonability.

### Results of feature ranking algorithms

According to **Fig 2(A)**, three feature-ranking algorithms (LightGBM, XGBoost, and CATBoost) were applied to the vibration and noise data one by one. Three feature lists, including LightGBM, XGBoost, and CATBoost feature lists, were obtained, which are provided in the S2 Table.

**Fig 3:**
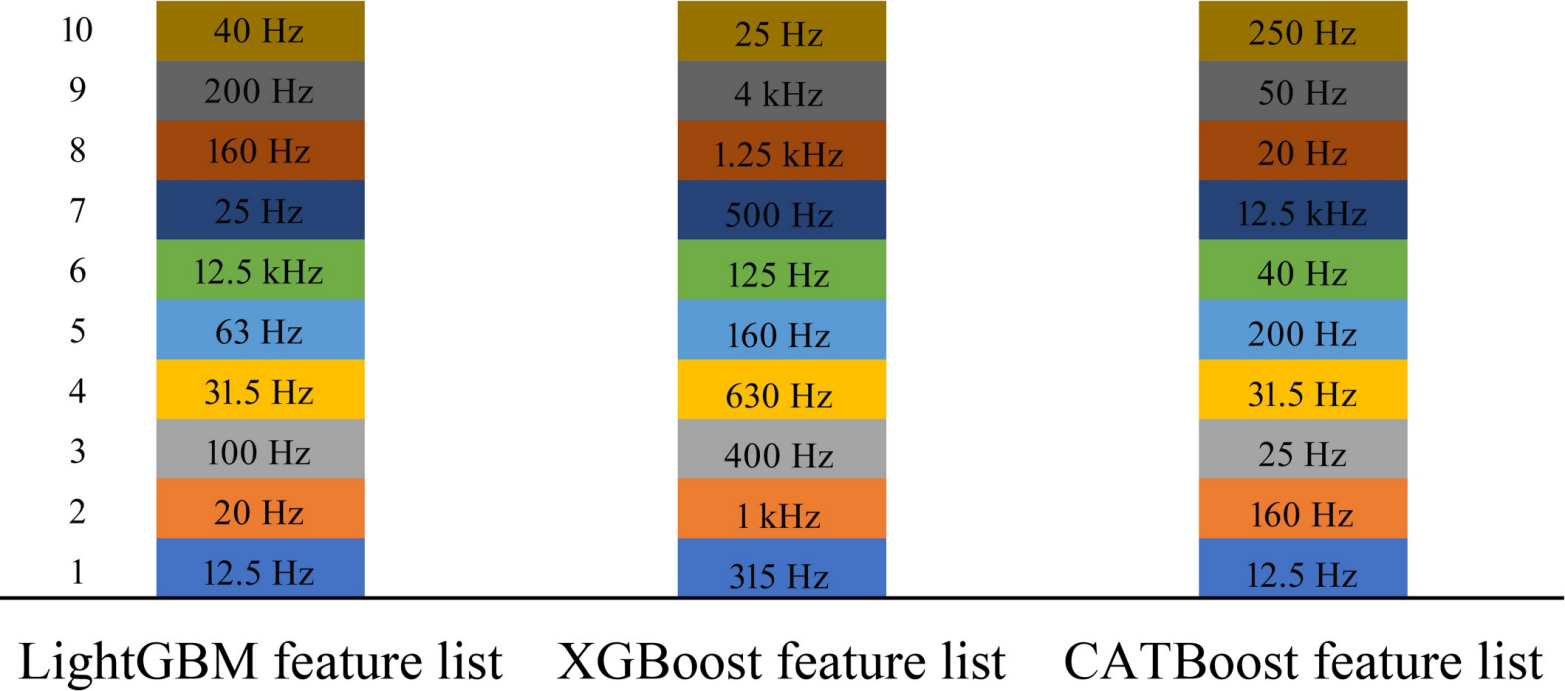
Bar chart to show the top ten features in three feature lists. The left numbers represent ranks in three feature lists. The top feature in LightGBM and CATBoost feature lists is “12.5 Hz”, whereas the top feature in XGBoost feature list is “325 Hz”. Differences exist between three feature lists, suggesting three feature ranking algorithms can evaluate the importance of features from different aspects.

**Fig 4:**
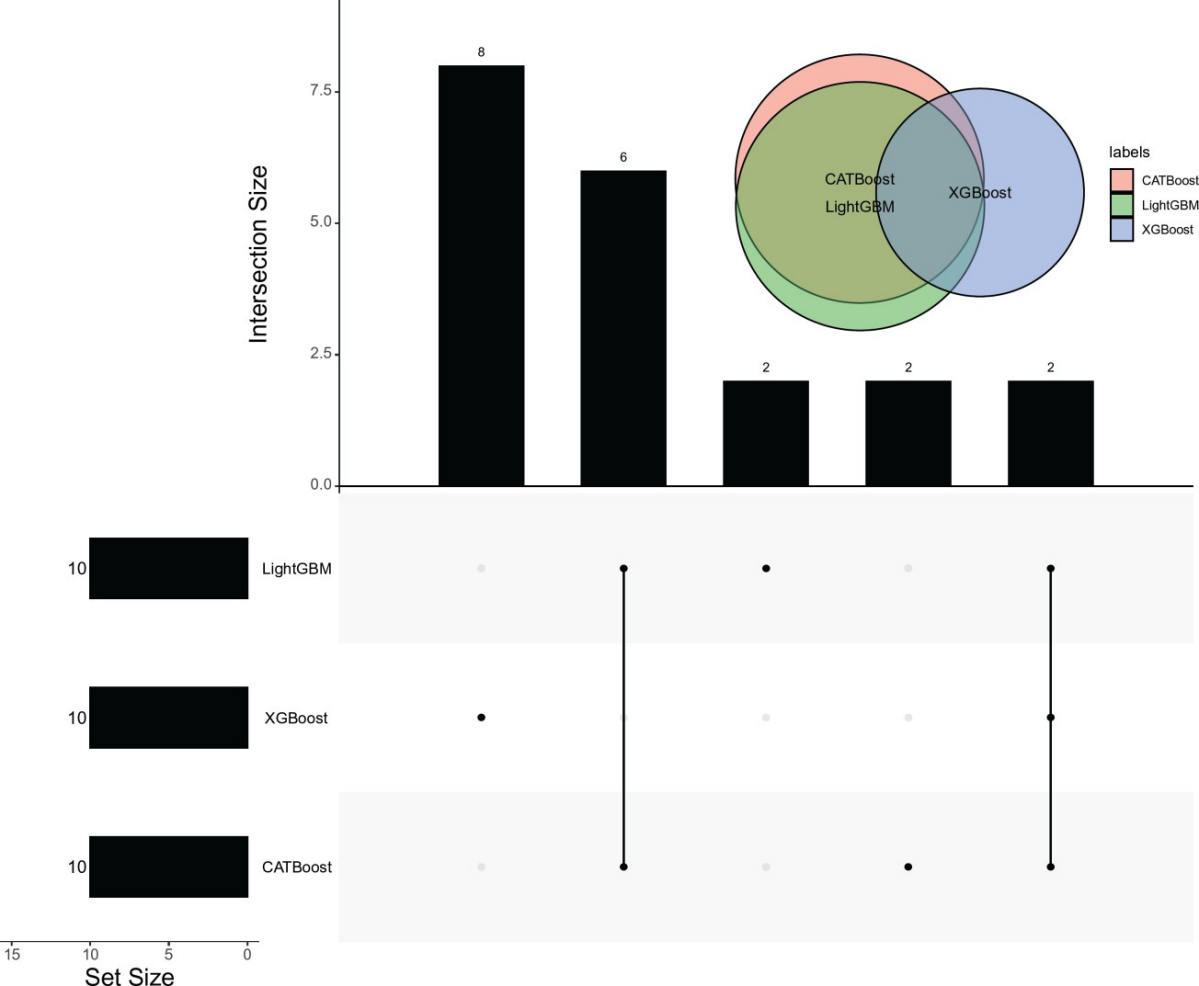
Upset graph to show the intersection of top ten features yielded by three feature ranking algorithms (LightGBM, XGBoost, and CATBoost). The top ten features yielded by XGBoost are quite different from those yielded by LightGBM and CATBoost. Each algorithm can find out exclusive features, suggesting that using multiple algorithms is helpful to mine important features as complete as possible.

At a glance, these three feature lists were not the same. It was reasonable because these algorithms investigated the data from different aspects. Top 10 features in each list were picked up for further analysis, which is shown in **Fig 3**. An observation is that “12.5 Hz” was the top feature in the LightGBM and CATBoost feature lists, whereas “315 Hz” was the top feature in the XGBoost feature list. The second, third, and fourth features (“1 kHz,” “400 Hz,” and “630 Hz”) in the XGBoost feature list were not in the top 10 features of the LightGBM and CATBoost feature lists, suggesting the significant differences between the XGBoost feature list and other two feature lists. As for the LightGBM and CATBoost feature lists, the second feature (“20 Hz”) in the LightGBM feature list was assigned the eighth place in the CATBoost feature list, and the third feature (“100 Hz”) was not in the top 10 features in the CATBoost feature list. Differences also existed between the LightGBM and CATBoost feature lists. Moreover, the top 10 features in three feature lists comprised three feature subsets. An upset graph was plotted to illustrate the intersection of these three feature subsets, as shown in **Fig 4**. The top 10 features discovered by XGBoost were quite different from those of LightGBM and CATBoost, consistent with the above analysis. In contrast, the top 10 features discovered by LightGBM and CATBoost were more similar because they commonly discovered eight features. However, each algorithm can discover exclusive features. In detail, XGBoost exclusively discovered eight features, and this number for LightGBM and CATBoost was two. These results suggested that three algorithms can mine common vital features as well as discover exclusive essential features, which was helpful in finding out all necessary features.

### Results of label removing test

As mentioned above, features related to one label cannot be extracted only based on three feature lists. Thus, a label-removing test was performed, thereby obtaining related frequencies for each deck. Three feature lists were generated when one label and samples under this label were removed, which are available in the S3 Table. Then, the rank gaps (**Eq 8**) were calculated for each feature. As three feature ranking algorithms were adopted in this study, each feature was assigned three rank gaps when one label was removed. Accordingly, three feature lists were generated with the increase in rank gaps of all features for each label, which are given in the S4 Table. The threshold *T* was set to 0.1, 0.2, 0.3, and 0.4 to select features related to one label from each of the three feature lists. For these threshold values, a small threshold (e.g., 0.1 or 0.2) may lead to the fact that no common features can be extracted from three feature lists. In this case, limited meaningful results can be obtained. A large threshold (e.g., 0.4) can bring too many common features, which may contain several false positive features. Thus, we finally set this threshold to a median value (0.3). Under this threshold, nine labels (decks) can obtain common related features (frequencies). These common features are provided in **Table 2**. Except for the 10th deck, each deck was assigned one or two related frequencies suitable for further analysis.

**Table 2:** Frequencies related to ten decks yielded by label removing test.

Deck	Number of frequencies	Frequency
Third deck	2	40 Hz, 80 Hz
Fourth deck	1	160 Hz
Fifth deck	2	200 Hz, 8 kHz
Sixth deck	1	31.5 Hz
Seventh deck	2	80 Hz, 1.25 kHz
Eighth deck	2	8 kHz, 5 kHz
Ninth deck	1	25 Hz
Tenth deck	0	-
Eleventh deck	1	80 Hz
Twelfth deck	1	1.25 kHz

### Results of the IFS method

**Fig 5:**
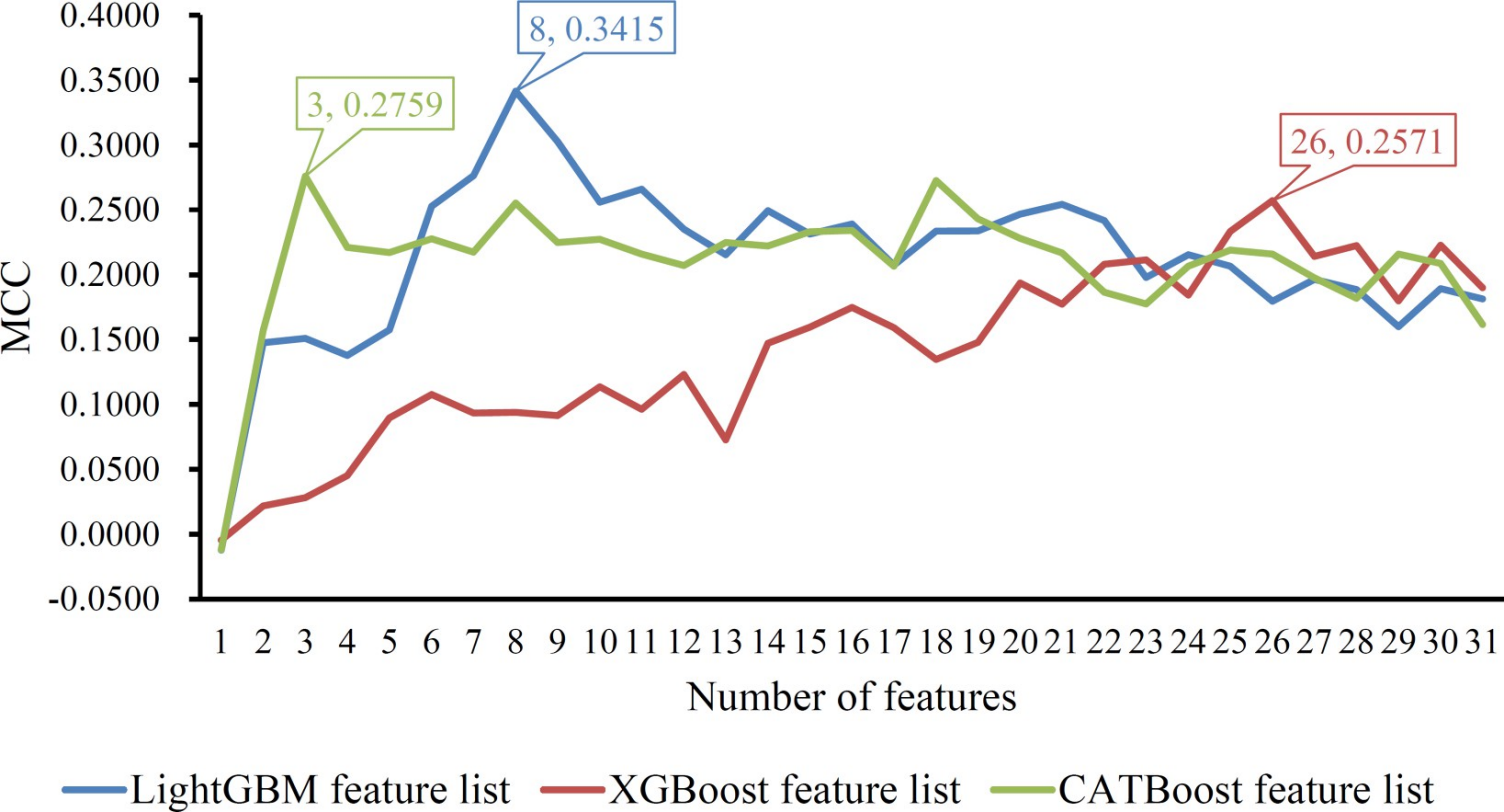
IFS curves on three feature lists. The Y-axis represents Matthews correlation coefficient (MCC) and X-axis represents the number of used features. The highest MCC is marked on each curve, along with the number of used features.

The other purpose of this study was to build a classifier to classify noise samples. Given a feature list, a series of classifiers with some top features in the list were set up and evaluated by five-fold cross-validation. The predicted results were counted as accuracy and MCC, which are provided in the S5 Table. For easy observation, an IFS curve was plotted for the IFS results on each feature list, where the MCC was set as the Y-axis, and the number of used features was defined as the X-axis. **Fig 5** shows the IFS curves. The highest MCC on the three curves were 0.3415, 0.2571, and 0.2759. Such performance was obtained by using the top 8, 26, and 3 features in LightGBM, XGBoost, and CATBoost feature lists, respectively. Accordingly, three optimal classifiers were built using these top features. Their detailed performance, including ACC, MCC, macro recall, and macro precision, is listed in **Table 3**. The accuracies of these classifiers were 0.4258, 0.3544, and 0.3698. Macro recall values were 0.3110, 0.2553, and 0.2767, whereas macro precision values were 0.2584, 0.2051, and 0.2258. Among these optimal classifiers, the classifier on the LightGBM feature list performed the best, and it can be used as a tool to classify noise samples. Although its performance was not very high, it was better than the results yielded by the random guess, which generated an accuracy of 0.1 as 10 labels were considered.

**Table 3:** Performance of the optimal classifiers on each feature list.

Feature list	Number of features	Accuracy	Matthews correlation coefficient	Macro recall	Macro precision
LightGBM feature list	8	0.4258	0.3415	0.3110	0.2584
XGBoost feature list	26	0.3544	0.2571	0.2553	0.2051
CATBoost feature list	3	0.3698	0.2759	0.2767	0.2258

**Fig 6:**
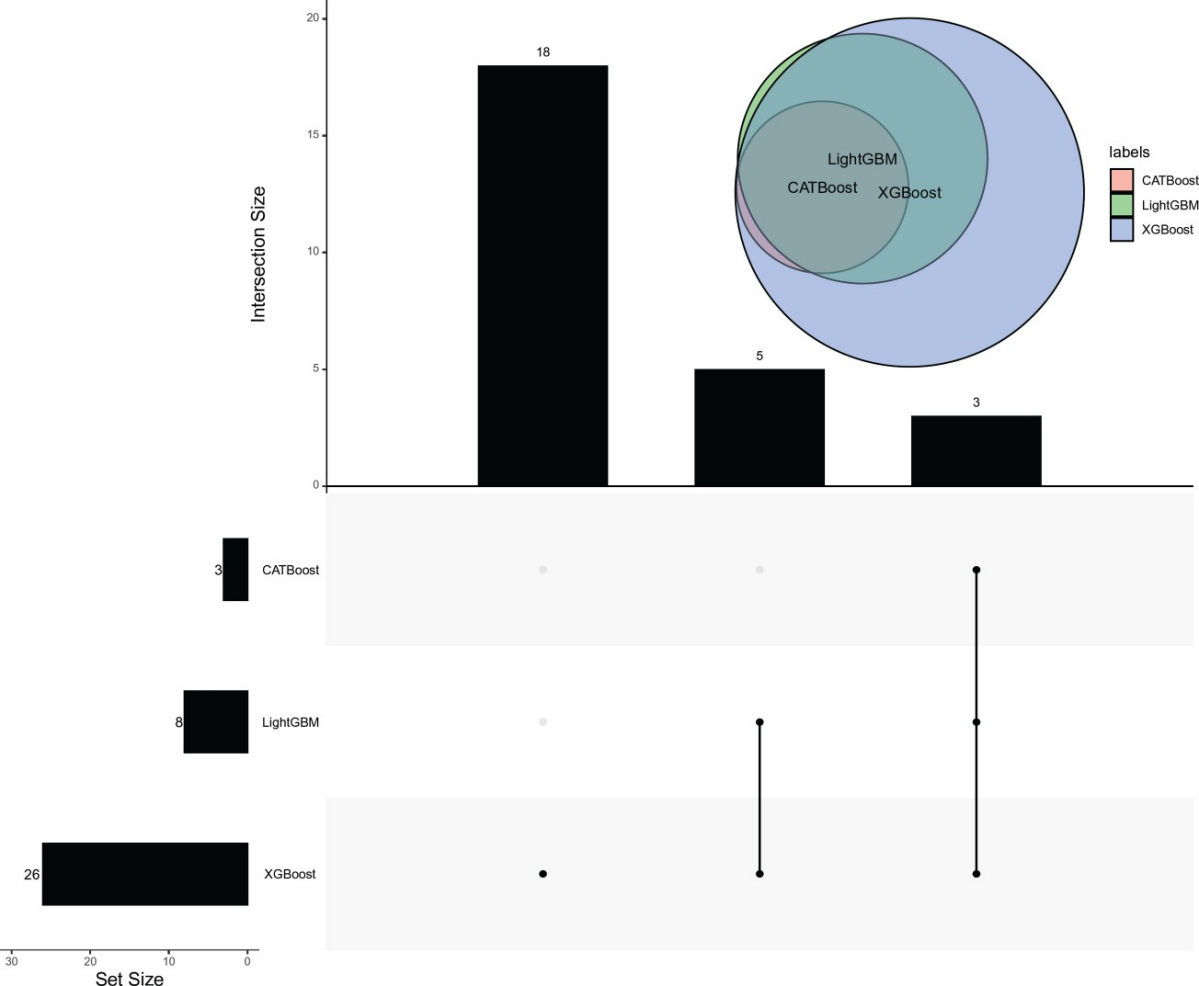
Upset graph to show the intersection of features used in three optimal classifiers. The features used in the optimal classifiers on the CATBoost and LightGBM feature lists are all used in the optimal classifier on the XGBoost feature list.

In addition, we picked up the features used in the above three optimal classifiers. An upset graph was plotted to display the intersection of these three feature subsets, as illustrated in **Fig 6**. As 26 features were used in the optimal classifier on the XGBoost feature list, occupying 83.87% of total features, they included the features used in the other two optimal classifiers. Furthermore, the features used in the optimal classifier on the CATBoost feature list were also used to construct the optimal classifier on the LightGBM feature list. These results indicated that the top three features (“12.5 Hz,” “160 Hz,” and “25 Hz”) in the CATBoost feature list may be the most important for classifying noise samples because all optimal classifiers used these features.

### Validating the results by statistical methods

**Table 2** lists the identified frequencies related to some decks. This section adopted the traditional statistical methods to show these findings were reasonable.

**Fig 7:**
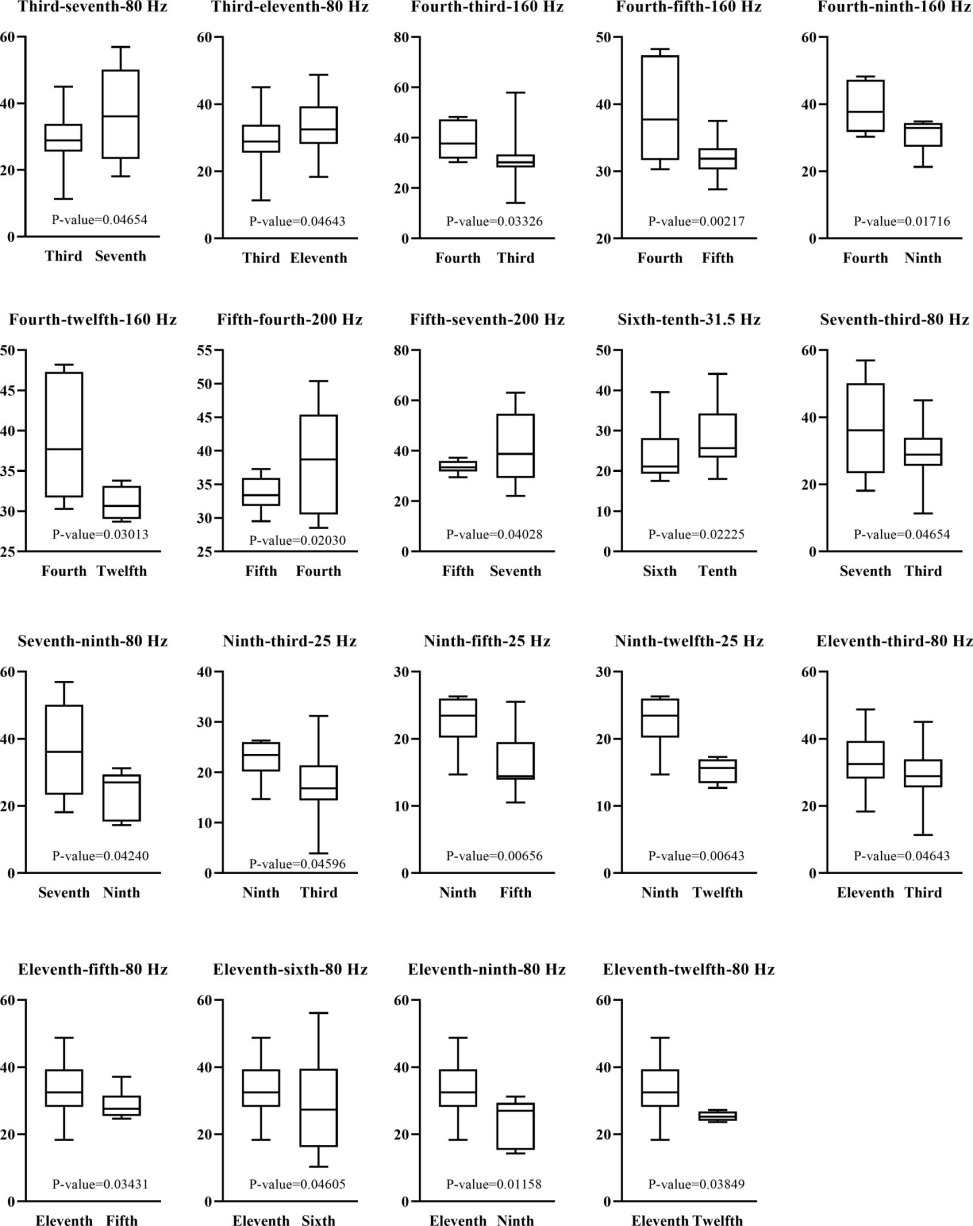
Box plot to show the significant differences between frequencies related to some decks and the same frequencies on other decks. The title of the subfigure consists of three parts. The first two parts indicate decks and the last part represent frequency. The third part is the frequency related to the first part. Under the same frequency, the first and second parts (decks) display significant differences. The p-value is lower than 0.05.

For each frequency related to a particular deck, the values were picked up under this frequency and divided into 10 groups based on 10 decks. After that, the differences between the values under the above deck and other decks were investigated by the student t-test, yielding nine p-values. The results indicated significant differences (measured by p-value<0.05) between some decks. For example, “80 Hz” was an important frequency related to the third deck. The data under this frequency on the third deck displayed a significantly different distribution from that on the seventh deck, with a p-value of 0.04654. Nineteen pairs of decks were obtained, as shown in **Fig 7**, where the box plot was placed to show the distribution of data under two decks, along with the p-value between two data groups. The above results indicated that some essential differences in frequencies between different decks that traditional statistical methods can obtain can also be discovered by the machine learning-based method. Furthermore, the other essential information may be mined using the machine learning-based method. As shown in **Fig 7**, the involved frequencies were all low. The traditional statistical methods discovered no high frequencies. However, some high frequencies were mined using the machine learning-based method, such as “8 kHz” for the fifth deck. These new findings were exclusively found using the machine learning-based method, suggesting that this method can be an important supplement for analyzing the vibration and noise data.

### Ablation tests

**Fig 8:**
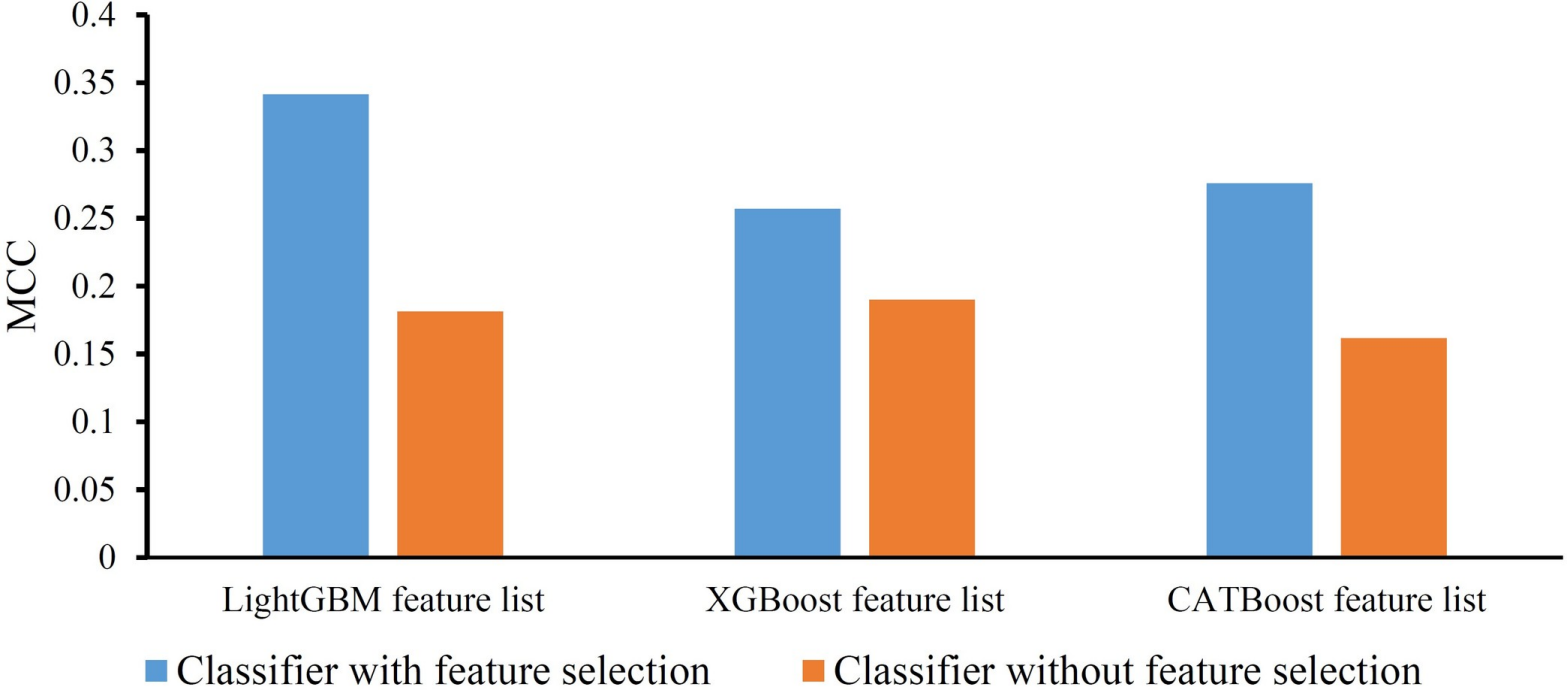
Bar chart to show the performance of classifiers with or without feature selection. Clearly, the classifiers with feature selection are better than those without feature selection.

This section contained an ablation test to confirm the important roles of feature ranking algorithms. All three ranking algorithms were removed; that is, all 31 features were fed into RF to construct the classifiers. These classifiers were also evaluated by five-fold cross-validation. Their performance (MCC) is displayed in **Fig 8**. For easy comparisons, the performance of optimal classifiers (i.e., classifiers with feature selection) is also shown in this figure. Evidently, the optimal classifiers provided better performance than the classifiers without feature selection. The advantage was approximately 16% (LightGBM feature list), 6% (XGBoost feature list), and 11% (CATBoost feature list). The feature ranking algorithms can provide positive contributions to constructing efficient classifiers in identifying vibration and noise samples.

In addition, the classification algorithm RF was replaced with a support vector machine (SVM) [34] to construct efficient classifiers. The best performance (MCC) of SVM on each feature list is listed in **Table 4**. For easy comparison, the best performance of RF on each feature list is also provided in this table. The RF provided a higher performance on the LightGBM feature list, whereas the SVM gave a better performance on the other two lists. However, RF accessed the best performance on the LightGBM feature list. Thus, RF was more appropriate for serving as the classification engine for the identification of vibration and noise samples.

**Table 4:** Comparison of the procedures with random forest and support vector machine.

Feature list	Classification algorithm	Number of features	MCC
LightGBM feature list	Random forest	8	0.3415
	Support vector machine	25	0.3173
XGBoost feature list	Random forest	26	0.2571
	Support vector machine	31	0.3006
CATBoost feature list	Random forest	3	0.2759
	Support vector machine	16	0.3302

### Limitations of this study and future work

Although the machine learning-based method successfully extracted frequencies related to some decks and built a classifier to distinguish noise samples, some limitations still exist. First, the extracted frequencies for some decks had not been confirmed by realistic scenes; that is, the reason why the frequencies and decks had strong relationships was unclear. This study only focused on the data analysis. Further deep mining can increase the practical value of this study. Second, the performance of the best classifier was far from excellent, which may be caused by the incompleteness of the vibration and noise data. If more noise samples were employed, we believed that more powerful classifiers could be constructed. Third, the investigated dataset was imbalanced. This study did not employ related methods to tackle this problem. By using some over-sampling or under-sampling methods, more powerful and balanced classifiers can be obtained. Finally, the proposed method adopted three feature ranking algorithms. Whether these algorithms were enough to mine all important features is unclear.

In the future, we will continue this work to overcome the above limitations, thereby designing more perfect methods. In recent years, deep learning algorithms, such as convolutional neural networks and recurrent neural networks, have been successfully applied to investigate audio data. However, their applications in vibration and noise data on ships were limited, which may be a new research direction in this area and can be used to design new methods for overcoming the above limitations.

## Conclusions

This study proposed a machine learning-based method to analyze the data on vibration and noise in cruise ships. Through this method, the special frequencies related to some decks were extracted. From these frequencies, the essential differences in vibration and noise among different decks can be obtained. The discovered frequencies also provided a clear direction to remove or reduce improper noise, thereby improving the comfort of cruise ships. Furthermore, a classifier was constructed to categorize noise samples through our method, enhancing the intelligence level of large cruise ships. Finally, this study adopted several newly proposed machine learning algorithms, providing an alternative way to investigate the vibration and noise data in cruise ships.

## Supporting information

S1 Table Data of vibration and noise collected from Adora Magic City. (XLSX)

S2 Table Three feature lists yielded by LightGBM, XGBoost, and CATBoost. (XLSX)

S3 Table Feature lists of ten decks when each label (deck) and samples with this label are removed. (XLSX)

S4 Table Feature lists of ten decks produced by the increasing in rank gaps of features. (XLSX)

S5 Table IFS results on three feature lists. (XLSX)
